# Quality by Design Methodology Applied to Process Optimization and Scale up of Curcumin Nanoemulsions Produced by Catastrophic Phase Inversion

**DOI:** 10.3390/pharmaceutics13060880

**Published:** 2021-06-15

**Authors:** Sandeep Kumar Reddy Adena, Michele Herneisey, Eric Pierce, Paul R. Hartmeier, Suneera Adlakha, Marco A. I. Hosfeld, James K. Drennen, Jelena M. Janjic

**Affiliations:** 1 Graduate School of Pharmaceutical Sciences, Duquesne University, Pittsburgh, PA 15228, USA; adenas@duq.edu (S.K.R.A.); herneiseym@duq.edu (M.H.); piercee1@duq.edu (E.P.); hartmeierp@duq.edu (P.R.H.); adlakhas@duq.edu (S.A.); hosfeldm@duq.edu (M.A.I.H.); drennen@duq.edu (J.K.D.); 2 Chronic Pain Research Consortium, Duquesne University, Pittsburgh, PA 15228, USA

**Keywords:** nanoemulsion, catastrophic phase inversion, quality by design, multiple linear regression

## Abstract

In the presented study, we report development of a stable, scalable, and high-quality curcumin-loaded oil/water (o/w) nanoemulsion manufactured by concentration-mediated catastrophic phase inversion as a low energy nanoemulsification strategy. A design of experiments (DoE) was constructed to determine the effects of process parameters on the mechanical input required to facilitate the transition from the gel phase to the final o/w nanoemulsion and the long-term effects of the process parameters on product quality. A multiple linear regression (MLR) model was constructed to predict nanoemulsion diameter as a function of nanoemulsion processing parameters. The DoE and subsequent MLR model results showed that the manufacturing process with the lowest temperature (25 °C), highest titration rate (9 g/minute), and lowest stir rate (100 rpm) produced the highest quality nanoemulsion. Both scales of CUR-loaded nanoemulsions (100 g and 500 g) were comparable to the drug-free optimal formulation with 148.7 nm and 155.1 nm diameter, 0.22 and 0.25 PDI, and 96.29 ± 0.76% and 95.60 ± 0.88% drug loading for the 100 g and 500 g scales, respectively. Photostability assessments indicated modest loss of drug (<10%) upon UV exposure of 24 h, which is appropriate for intended transdermal applications, with expected reapplication of every 6–8 h.

## 1. Introduction

Curcumin (CUR) is a hydrophobic, polyphenolic phytoconstituent extracted from the turmeric plant (*Curcuma longa*) [1]. Over the past 50 years, CUR has received growing interest in the pharmaceutical industry due to its anti-oxidant, anti-inflammatory, anti-diabetic, anti-hyperlipidemic, wound healing, hepatoprotective, anti-tumor, anti-mutagenic, anti-arthritic, anti-microbial, anti-parasitic, and anti-viral properties and avails in the treatment of many ailments, including Crohn’s disease, liver cirrhosis, ulcerative colitis, Alzheimer’s disease, rheumatoid arthritis, heart attacks, peptic and gastric ulcer, cancer, psoriasis, and vitiligo [2,3,4,5,6,7,8]. CUR is a natural anti-oxidant capable of scavenging a wide range of oxidative stressors, including superoxide radicals, hydrogen peroxide, and ferrous ions [9]. CUR can reduce inflammation through the improvement of oxidative markers [10] and has innate anti-inflammatory properties. CUR is generally regarded as safe (GRAS) by the FDA, with oral doses of up to 12 g being well-tolerated, and is widely available as an over-the-counter supplement [3,5,11]. Despite its promising therapeutic benefits and pharmacological safety, the clinical efficacy of curcumin-based formulations is limited due to low relative bioavailability (<1%), low aqueous solubility (7 µg/mL), lipophilicity (LogP = 3.2), high metabolic rates in the intestine and liver, and rapid excretion from the body [12,13,14,15,16,17,18]. CUR exists naturally in only one crystalline form and is slightly hygroscopic (0.2% *w*/*w* water adsorption at 90% relative humidity). CUR is photolabile, and in solution, it degrades rapidly at 37 °C/pH 7.2, further complicating the formulation efforts [13,15,19]. Despite these challenges, CUR continues to be a molecule of interest due to its therapeutic benefits, and a variety of formulations have been examined in the literature, spanning from nanoemulsions, microemulsions, solid particles, hydrogels, and co-crystals [2,6,20,21,22,23,24].

Nanoemulsions offer a clear solution to the low water solubility by confining CUR in an oil phase during delivery. Further, this sequestering of CUR to oil phases reduces the potential for crystallization and improves stability even at accelerated conditions [6,23,25,26]. Finally, nanoemulsions are capable of bypassing the gastrointestinal tract through transdermal delivery and localized delivery to specific sites [27]. Nanoemulsions increase the flux of hydrophobic drugs across the subcutaneous bilayer through several proposed mechanisms, including disruption of the subcutaneous lipid bilayer, enhanced permeation due to reduced droplet size, electrostatic interactions between the skin and the nanoemulsions, and increased permeability through viscosity reduction of the nanoemulsion [27]. CUR-loaded nanoemulsions and microemulsions have been shown previously to improve flux across membranes [6,28,29].

Nanoemulsions are colloidal dispersions with droplet diameters of less than 500 nm. Droplet formation requires the addition of a surfactant or combination of surfactants, amphiphilic compounds that lower the interfacial energy between the hydrophobic and hydrophilic phases [30,31,32]. Droplet formation is dependent on the Laplace pressure, which is a function of the droplet size and the interfacial tension between the oil and aqueous phases. Thus, small particle sizes (<500 nm) can be achieved by (1) lowering the interfacial tension through increasing surfactant content or (2) overcoming the Laplace pressure through high energy manufacturing processes. High energy processes, such as high-pressure homogenization (shear pressure) and sonication (sound pressure) are commonly used to achieve the pressure gradients necessary for droplet formation. These methods require highly specialized equipment capable of achieving the energy input and thus may not be preferable for manufacturing pharmaceuticals, due to high up-front investment in equipment or reliance on contract manufacturing organizations [31].

In contrast, low energy methods rely on decreasing the interfacial tension through an increase in surfactant content such that the droplets can form without applying considerable shear or pressure to the system [31]. These methods require fewer processing steps, do not require specialized equipment, and can be performed using simple magnetic stirring for mixing, with or without heating. The three main processes of formation for nanoemulsions using low energy methods are: (1) spontaneous emulsification (SE), (2) temperature phase inversion (PIT), and (3) catastrophic phase inversion (CPI). Spontaneous emulsification is achieved by adding an oil phase into the aqueous phase with mixing, where droplets form through the collapse of the hydrophobic oil into a shell of surfactant. The surfactant will generally be dispersed into the oil phase prior to the titration into the aqueous phase, but a fraction can be pre-dispersed into the aqueous phase [30,32,33]. PIT and CPI both function by changing the curvature of the surfactant interface between oil and water, which transitions from w/o emulsions to o/w emulsions through a bicontinuous phase, where the curvature of the micelles reaches 180°. PIT achieves droplet formation by increasing the temperature, which changes the affinity of the surfactants for the aqueous phase, forming the w/o emulsion, followed by cooling to cause the inversion [32]. A major limitation in PIT is that the formation of the o/w emulsion and the resultant droplet size is dependent on the cooling rate during the inversion. For commercial-scale manufacturing, cooling large volumes in a controlled manner presents significant challenges, limiting the viability of PIT for pharmaceutical products. CPI achieves the inversion of the surfactant angle through changes in the composition of the system in a controlled manner. Oil and surfactant are mixed, and then the aqueous phase, with or without additional surfactant, is titrated into the oil phase, forming the initial w/o emulsion. As the water is titrated into the system, the surfactant(s) become more hydrated and transition to the bicontinuous phase, where the curvature is zero. Upon further titration, the curvature inverts and the o/w emulsion is formed [30,31,33,34]. CPI is preferable to PIT for large-scale manufacturing because it can be performed at ambient temperature, which allows for a more robust and scalable process.

**Figure 1:**
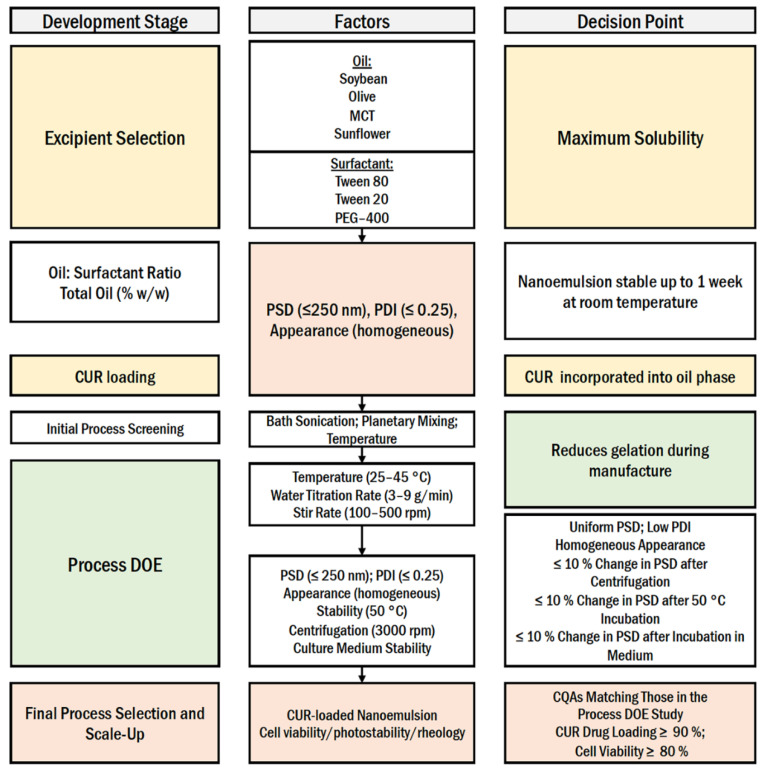
The quality by design (QbD) approach for the process parameters optimization and product quality control for a curcumin nanoemulsion produced by catastrophic phase inversion low-energy emulsification.

To date, a variety of CUR nanoemulsions have been formed using high energy methods, but there is limited information on simple, low-energy manufacturing methods for CUR formulations [20,21,22,23,24,25]. Here, we report a low-energy manufacturing scheme for the production of a CUR nanoemulsion using CPI. A quality by design (QbD) approach was used as the template for the development process. Briefly, QbD is a development process defined by the International Council for Harmonization (ICH) Q8 as a “systematic approach to development that begins with predefined objectives and emphasizes product and process understanding and process control, based on sound science and quality risk management”. Figure 1 summarizes our presented approach for applying QbD to the process optimization of a low energy CUR nanoemulsion. This approach consisted of stepwise development stages, each stage containing predefined decision points that had to be met prior to proceeding to the next development stage. During the first development stage (excipient selection), oils and surfactants were screened for CUR solubility. The oil/surfactant combination that maximized CUR solubility proceeded to preliminary process screening, where nanoemulsion composition (oil:surfactant ratio and total oil content (% *w*/*w*)) was investigated to (1) confirm the formation of the nanoemulsion via CPI, (2) identify a nanoemulsion that maintained colloidal stability upon 1 week of storage at ambient temperature, and (3) verify that the select formulation solubilized CUR at the predefined target concentration (0.1% *w*/*w*). The resultant formulation exposed a system that undergoes CPI through a liquid crystalline intermediate (gel phase), which presented challenges during manufacturing. To mitigate these challenges, a risk analysis was used to develop a design of experiments (DoE) that investigated the impact of manufacturing process parameters on the formation of the gel phase and final product diameter, PDI, and colloidal stability. Results from the DoE were used to identify the optimal manufacturing process parameters that minimized the nanoemulsion droplet diameter while maximizing nanoemulsion colloidal stability. These optimal manufacturing process parameters were then used to successfully produce CUR-loaded nanoemulsions on 100 g and 500 g scales. Scale-up to 500 g did not significantly impact nanoemulsion droplet diameter, PDI, drug loading, or colloidal stability. Ultimately, this work provides the basis for producing a simple, scalable low-energy nanoemulsion with the potential to improve CUR delivery.

## 2. Materials and Methods

### 2.1. Materials

Caprylic/capric triglyceride (MCT oil, C3465), olive oil (OL130), soybean oil (S0255), polyethylene-glycol 400 (P0110), and Tween 80 (P0138) were purchased from Spectrum Chemical (New Brunswick, NJ, USA). Sunflower oil (L200870P) was purchased from Spectrum Naturals (Petaluma, CA, USA). Tween 20 (W291501) and curcumin (C1386) were purchased from Sigma–Aldrich (St. Louis, MO, USA).

### 2.2. Curcumin Solubility in Oils and Surfactants

CUR solubility was evaluated in several oils (soybean, olive, MCT, and sunflower) and surfactants (Tween 20, Tween 80, and polyethylene glyocol-400 [PEG-400]). CUR was weighed into 2 mL microcentrifuge tubes at 50 mg for oils and 100 mg for surfactants. In total, 1 mL of oil or surfactant was added to each tube and vortexed for approximately 1 min to create a slurry. Slurries were covered with foil to protect from light and shaken at 1000 rpm for 3 days. After 3 days, samples were centrifuged at 15,000 rpm for 10 min. A total of 100 µL of supernatant was diluted 100× into 9.9 mL of ethanol and vortexed to solubilize and prevent crashing out of CUR. The ethanol solutions were analyzed by UV-Vis (HP8453, Agilent, Waldbronn, Germany) using a 10.00 mm pathlength glass cuvette from 200 to 600 nm wavelength. The samples were further diluted prior to analysis to final dilutions of 200× for oils and 10,000× or 20,000× for surfactants in ethanol. A standard curve was constructed by diluting a 100 µg/mL curcumin stock solution in ethanol to 8 µg/mL and then serially diluting to 4, 2, 1, and 0.5 µg/mL in ethanol. The samples were analyzed at 425 nm to determine CUR concentration.

### 2.3. Design of Experiments and Multiple Linear Regression Modeling

A design of experiments (DoE) was constructed using JMP Pro 14 software (SAS Institute, Cary, NC, USA) to determine the impact of nanoemulsion processing parameters on nanoemulsion diameter and gelation. Parameters to be evaluated were selected based on the results of the risk assessment analysis (see Supplementary Materials) and the process development screening studies. These included water titration rate (3 and 9 g/min), stir rate (100 and 500 rpm), and temperature (25 and 45 °C). The selected parameters were used to develop a 2-level, 3-factor full factorial design of experiments (DoE) with three center points. A summary of this design is provided in Table 1.

Nanoemulsions produced as a part of the DoE were evaluated for droplet diameter, polydispersity index (PDI), and colloidal stability. Colloidal stability was evaluated by observing changes in the nanoemulsion droplet diameter and PDI in response to a variety of stressors (centrifugation, incubation in serum, and incubation at elevated temperature). Details regarding colloidal stability assessment are provided in Section 2.5. The DoE results were used to construct an MLR model capable of predicting nanoemulsion diameter as a function of nanoemulsion processing parameters (JMP Pro, v14, SAS Institute, Cary, USA). All one-way and two-way interaction terms were included in the full model and removed stepwise such that only terms with a *p*-value < 0.05 were included in the final model.

### 2.4. Dynamic Light Scattering

Droplet diameter and polydispersity index (PDI) were determined using DLS (Zetasizer NanoZS, Malvern, UK), as reported previously [35]. Nanoemulsions were diluted in disposable cuvettes at 1:80 *v/v* in de-ionized water to a total volume of 1 mL. Droplet diameter and polydispersity index (PDI) were measured in triplicate at 25 °C and at a light scattering angle of 173°.

### 2.5. Nanoemulsion Stability Testing

#### 2.5.1. Centrifugal Force Stability

The undiluted nanoemulsion (1.0 mL) was centrifuged using Centrifuge 5804R (VWR; Eppendorf AG, Hamburg, Germany) in a 15 mL plastic centrifuge tube at 3000 rpm for 5 min [36]. The centrifuged samples were diluted at 1:80 *v/v* in deionized water, and the droplet diameter and PDI of the nanoemulsions were measured by DLS.

#### 2.5.2. Serum Stability

Nanoemulsion was diluted at 1:80 *v/v* in biological media (20% FBS in DMEM) or in de-ionized water in a 1.5 mL Eppendorf tube. Droplet diameter and PDI were evaluated with DLS immediately after dilution (0 h time point) and after incubation at 37 °C for 72 h.

#### 2.5.3. Thermal Stability

Undiluted nanoemulsion (1.0 mL) was aliquoted to a 1.5 mL Eppendorf tube and placed in a small beaker containing a small volume of de-ionized water (to prevent nanoemulsion evaporation). The beaker was covered with aluminum foil and incubated at 50 °C for 7 days. After 7 days, incubated nanoemulsion was diluted at 1:80 *v/v* in de-ionized water. Droplet diameter and PDI were evaluated with DLS.

### 2.6. Drug Free Nanoemulsion Production

Nanoemulsions were prepared at a 100 g scale in a 250 mL glass beaker. Oil and surfactant were weighed into the beaker and mixed for 30 min with a Teflon magnetic stir bar at a specified stir rate (100, 300, 500 rpm) and temperature (25, 35, 45 °C). While keeping stir rate and temperature constant, water was titrated using a 100–1000 µL positive displacement pipette at a specified water titration rate (3, 6, 9 g/min). Gelation was typically observed after approximately 10 g of water was added. At this point, the product was mixed manually using a stainless-steel spatula, and water titration was continued at the specified rate until the stir bar could continue mixing. Upon completion of water titration, the product continued stirring at the specified stir rate and temperature for 90 min. Nanoemulsions were stored at ambient temperature (18–22 °C).

### 2.7. Manufacture and Scale-up of CUR-Loaded Nanoemulsion

CUR-loaded nanoemulsions were produced on 100 g and 500 g scales using an optimized processing method identified through DoE and MLR modeling. Specifically, CUR, MCT oil, and Tween 80 were weighed into a beaker (250 mL for 100 g scale, 1000 mL for 500 g scale) and mixed for 30 min at a stir rate of 100 rpm (100 g scale) or 300 rpm (500 g scale) and a temperature of 25 °C. Water titration was then performed at a rate of 9 g/min (100 g scale) or 45 g/min (500 g scale). During gelation, the product was mixed manually using a stainless-steel spatula until the magnetic bead on the stir bar could continue mixing. Upon completion of water titration, the final product continued to stir at 100 rpm (100 g scale) or 300 rpm (500 g scale) and at a temperature of 25 °C for 90 min. CUR-loaded nanoemulsions were evaluated for droplet diameter, polydispersity index, drug loading, and stability. Specifically, CUR nanoemulsion stability was assessed using the same criteria that were specified to evaluate the nanoemulsions produced in the 11-run DoE (centrifugation stability, serum stability, and 1-week incubation at 50 °C).

### 2.8. CUR Drug Content by High Performance Liquid Chromatography (HPLC)

CUR nanoemulsions were diluted into acetonitrile to a final concentration of 50 µg/mL CUR and then diluted into water to a final diluent composition of 50/50 acetonitrile/water (*v*/*v*). Standards were prepared likewise from 0.5 to 50 µg/mL final CUR concentration. Samples and standards were run using an isocratic HPLC method with a 1.2 mL/min flow rate, 33 °C column temperature, and 50 µL injection volume. The mobile phase was 60/40 acetonitrile/water (with 2% acetic acid) (*v*/*v*). The column was a 150 × 4.6 mm gold C18 column with a 5 µm pore size (Hypersil Gold C18, 25,005–154,630, Thermo Fisher, Vilnius, Lithuania).

### 2.9. Cell Viability

CellTiter-Glo^®^ luminescence assay was used to assess the cell viability of the CUR-loaded nanoemulsion [37]. Briefly, RAW 264.7 cells (mouse macrophages) were plated at 10,000 cells/well in a 96-well plate. After incubating overnight at 37 °C and 5% of CO_2_, culture media were separated. The adhered cells were exposed to different concentrations (0.31–40 µL of nanoemulsion/1 mL of media) of CUR-loaded nanoemulsion (prediluted in complete medium) and incubated for 6 h. Upon completion of the incubation, 100 µL of the medium was removed carefully and 40 µL of CellTiter-Glo^®^ (Promega, Madison, WI, USA) reagent was added to each well. To protect the samples from light, the plate was covered with aluminum foil, and to induce the cell lysis, the plate was shaken on Lab Doctor™ Orbital Shaker (Heidolph, Schwabach, Germany) for 20 min. Luminescence was recorded on a microplate reader (1420 Multilabel Counter, Victor3™; Perkin Elmer, Waltham, MA, USA).

### 2.10. Rheology

Rheological properties of the CUR nanoemulsions prepared at two levels of scale (100 g and 500 g) were tested for rheological behavior using a rheometer (DHR-20, TA Instruments, New Castle, DE, USA) equipped with parallel plate geometry (60 mm diameter, 300 µm gap). Amplitude oscillation testing and frequency oscillation testing were carried out on the 1 mL samples to determine the critical strain values and single amplitude across multiple frequencies, respectively. A single amplitude across multiple frequencies was used for the testing, from 0.1 rad/s–1 rad/s, a strain percentage of 100,000% was used, from 1 rad/s–10 rad/s, a strain percentage of 10,000% was used, and from 10 rad/s–100 rad/s, a strain percentage of 1000% was used [38].

## 3. Results

### 3.1. Excipient Selection and Formulation Development

Nanoemulsions developed via catastrophic phase inversion (CPI) are an attractive option for the delivery of lipophilic drugs, such as curcumin (CUR). Nanoemulsions have the potential to improve CUR solubility, protect CUR from temperature and/or light-induced chemical destabilization, and increase CUR bioavailability through avoidance of first pass metabolism. Stable nanoemulsions can be developed using relatively low concentrations of surfactants (compared to microemulsions), potentially improving the safety of these formulations. However, this typically necessitates the use of high energy equipment (e.g., microfluidization, sonication) that can complicate scale-up efforts. A CUR nanoemulsion that could be reproducibly developed via CPI could prove to be both safe, affordable, and scalable. Our group has previously utilized a quality by design (QbD) approach to investigate the impact of composition and manufacturing parameters on the diameter, PDI, drug loading, and stability of microemulsions [36] produced using a low energy manufacturing approach and nanoemulsions [39] produced using high energy approaches [40]. This report is our first exploration into CPI nanoemulsion formulation and stability.

**Figure 2:**
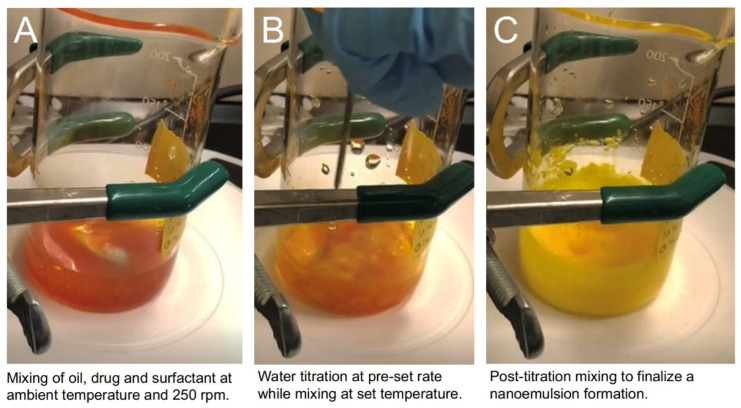
Manufacturing of nanoemulsions using the CPI low-energy emulsification method: (**A**) Pre-titration: mixing oil, surfactant, and drug; (**B**) water (aqueous phase) titration. As water is added, a viscous liquid crystalline gel is formed, requiring manual stirring; (**C**) Post-titration: curcumin loaded nanoemulsion is formed after continued stirring for 60–90 min.

During the development stage of excipient selection (Figure 1), CUR solubility was evaluated in several oils (soybean, olive, caprylic/capric triglycerides (MCT), sunflower) and surfactants (Tween 80, Tween 20, PEG-400). CUR exhibited the highest oil and surfactant solubility in MCT oil (2.4 mg/mL) and Tween 80 (83.4 mg/mL), respectively, which were selected for formulation and process development (Supplemental Figure S1). During the second development stage (Figure 1), nanoemulsion oil:surfactant ratio and total oil content (% *w*/*w*) were investigated to identify a formulation that maintained colloidal stability (determined by visual inspection and dynamic light scattering (DLS) measurements) for 1 week at ambient temperature. Specifically, MCT oil was examined at 10% (*w*/*w*) with 1:1 and 1:0.8 oil:surfactant ratios (SORs), 20% (*w*/*w*) at 1:1, 1:0.9, 1:0.8, and 1:0.75 SORs (Supplemental Table S1), following an earlier reported study [21]. All formulations exhibited CPI characterized by a rapid increase in viscosity when water titration began, resulting in a liquid crystalline gel phase. The accompanying viscosity increase stopped the magnetic stir bar, and mechanical assistance with a stainless-steel spatula was necessary to continue stirring. As the water was titrated into the system (approximately 30 g), the stir bar could continue to mix the system. However, the undissolved gel phase remained after water titration was complete, which necessitated stirring for an additional 90 min after water titration was completed. An example of the observed gelation during manufacturing is provided in Figure 2. After post-titration stirring, all emulsions were opaque, white (CUR-free), or yellow (CUR-loaded), free of visible solids with particle sizes less than 250 nm for all O/S ratios. This result is consistent with O/S ratios of successful emulsions previously manufactured [20,21,23,30,33,34].

**Figure 3:**
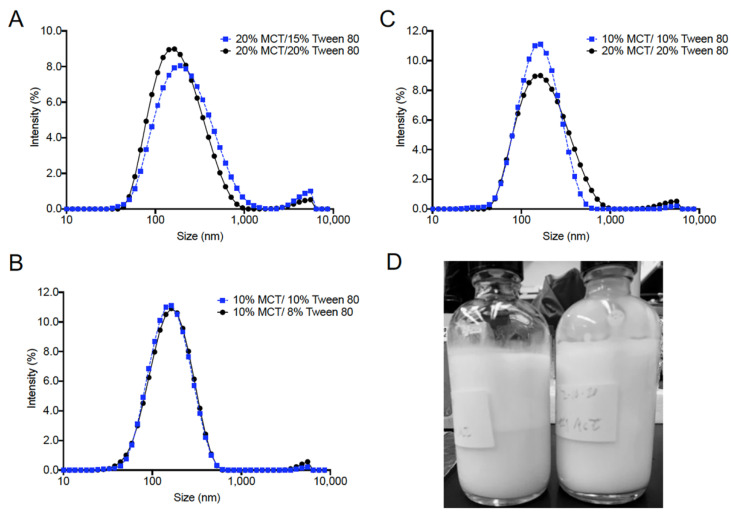
Nanoemulsions droplet size comparison for varied nanoemulsion compositions during pre-formulation studies. (**A**) 20% MCT oil/20% Tween 80 vs. 20% MCT oil/15% Tween 80; (**B**) 10% MCT oil/10% Tween 80 vs. 10% MCT oil/8% Tween 80; (**C**) 10% MCT oil/10% Tween 80 vs. 20% MCT oil/20% Tween 80 compositions; and (**D**) visual inspection of select nanoemulsions during pre-formulation studies. Image of typically observed phase separation after 1 week of storage at ambient temperature. These nanoemulsions are considered failed formulations.

**Figure 4:**
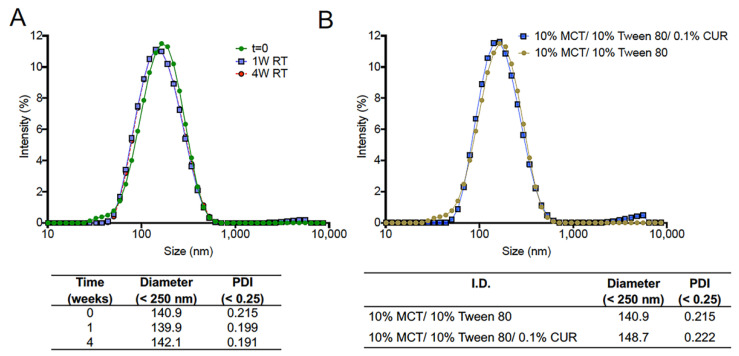
Size distribution follow up over time for nanoemulsions prepared during pre-formulation studies. (**A**) Droplet size and PDI comparison of 10% MCT oil/10% Tween 80 nanoemulsions up to 1 month at room temperature and (**B**) droplet size distribution comparison of 10% MCT oil/10% Tween 80 vs. 10% MCT oil/10% Tween 80/0.1% CUR nanoemulsions.

Key DLS and visual comparisons between the screening formulations are provided in Figure 3. At higher MCT oil content (20% *w*/*w*), increasing the SOR results in a decrease in nanoemulsion droplet diameter, from 180.8 nm to 154.7 nm, while the PDI was also reduced from 0.369 to 0.264 (Figure 3A). However, at lower MCT oil content (10% *w*/*w*), there does not appear to be a significant impact of SOR on initial (day 1) nanoemulsion droplet diameter, as shown in size distribution overlays (Figure 3B). Further, when SOR is held constant at 1.0, initial nanoemulsion droplet diameter remains constant, regardless of MCT oil content (Figure 3C). However, MCT oil content and SOR have a significant impact on long-term nanoemulsion stability. Nanoemulsions with high (20% *w*/*w*) MCT oil content and nanoemulsions with SOR < 1 exhibited phase separation within 1 week of production when stored at ambient temperature. An example of this phase separation is shown in Figure 3D. The right image is a colloidally stable nanoemulsion, and the left image is a nanoemulsion that has undergone phase separation. Based on these results, a composition of 10% *w*/*w* MCT oil and 10% *w*/*w* Tween 80 was selected to proceed to the next development stage (CUR-loaded nanoemulsion preparation). A nanoemulsion pair with and without CUR at 0.1% *w*/*w* was developed and evaluated for 4 weeks at ambient storage (Figure 4). Nanoemulsions maintained colloidal stability for 4 weeks when stored at ambient temperature, as determined by no significant change in nanoemulsion droplet diameter or PDI during the storage period. The size distribution overlays at 0, 1, and 4 week-time points are shown in Figure 4A. Incorporation of CUR at 0.1% *w*/*w* resulted in a small increase in nanoemulsion droplet diameter (~8 nm) and PDI (~0.07, Figure 4B). Incorporation of CUR did not impact nanoemulsion colloidal stability, and further, CUR remained fully solubilized in the nanoemulsion after 4 weeks of storage at ambient temperature (determined by visual inspection). Thus, this nanoemulsion formulation (10% *w*/*w* MCT oil, 10% *w*/*w* Tween 80) proceeded to the next development stage (initial process screening, Figure 1).

### 3.2. Initial Process Screening

The initial process screening study was performed to determine if the gelation observed in the SOR study could be controlled via changes in the nanoemulsion manufacturing process. Low-energy bath sonication was evaluated as a post-titration gel dispersal mechanism and as a primary mixing mechanism. A planetary mixer was also used as a mixing mechanism, replacing the presence of a stir bar. These methods were not able to reduce the observed gelation and were abandoned as potential process changes. Finally, water titration was performed at 50 °C, which reduced the apparent viscosity of the gel phase but did not remove it entirely. Although gelation complicates the manufacturing process, it does not appear to impact the final nanoemulsion product. Rather, the observed gelation may be a cubic liquid crystal intermediate phase that is a critical component for nanoemulsion formation [41]. Previous work has demonstrated that the time spent in the crystal intermediate phase prior to dilution is an important component of this transition [42]. Thus, water titration rate and stir rate may impact nanoemulsion droplet diameter, PDI, and colloidal stability, as these parameters could impact the length of time spent in the crystal intermediate phase. A risk analysis (see supplementary information) confirmed that temperature, water titration rate, and stir rate were most likely to impact nanoemulsion stability. Therefore, these parameters were further investigated in a process design of experiments (DoE).

### 3.3. Selection of Critical Quality Attributes and Design of Experiments

**Figure 5:**
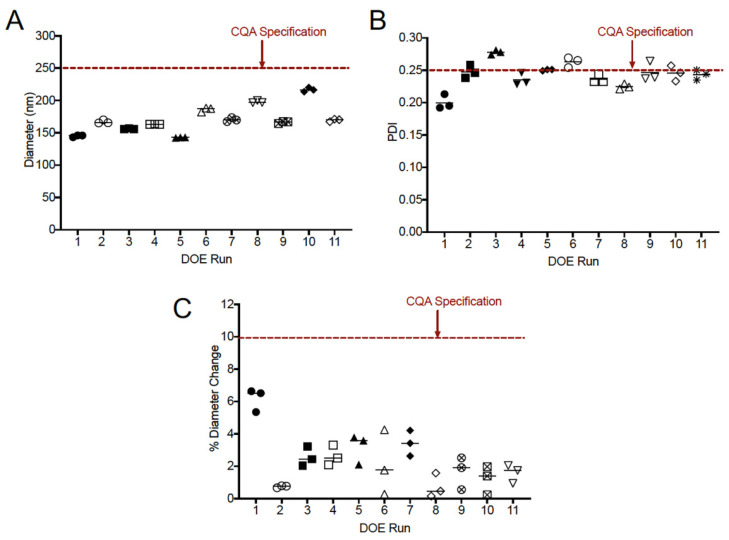
(**A**) Droplet diameter comparisons of the 11 DoE runs (each represented with its unique symbol); (**B**) PDI comparisons of the 11 DoE runs; and (**C**) % diameter change of DoE runs after storage for 1 week at 50 °C.

A 2-level, 3-factor full factorial design of experiments (DoE) with three center points was developed to determine the impact of stir rate, water titration rate, and temperature on nanoemulsion droplet diameter, PDI, colloidal stability, and observed gelation phenomena. The composition and manufacturing conditions for each run of the DoE are shown in Table 1. Composition for each run of the DoE was identical (10% *w*/*w* MCT oil, 10% *w*/*w* Tween 80), as the optimal composition was identified during the previous development stages. Two levels were selected for temperature (25 and 45 °C), titration rate (3 and 9 g/min), and stir rate (100 and 500 rpm), and all combinations of temperature, titration rate, and stir rate were evaluated (8 runs). The center point (35 °C, 6 g/min, 300 rpm) was also evaluated in triplicate to assess nanoemulsion reproducibility, bringing the total number of runs to 11. To facilitate rapid identification of a stable final product, extensive quality control analyses were performed for each run of the DoE. These criteria were defined as critical quality attributes (CQAs). CQAs are a part of the QbD approach and are defined as characteristics (physical, chemical, biological, etc.) that must fall within a specific range (CQA specification) to ensure final product quality. Nanoemulsion CQAs and specifications are provided in Table 2. These CQAs include appearance (homogenity), initial droplet diameter, and PDI, as well diameter change and PDI after (1) centrifugation at 3000 rpm, (2) incubation in serum containing media at 37 °C for 72 h, and (3) incubation at 50 °C for 7 days. Droplet diameter and PDI are important CQAs, as the target delivery mechanism (transdermal) is impacted by droplet size, with smaller nanoemulsion diameter and tighter control of droplet dispersity enabling more consistent performance [27]. Centrifugation and 50 °C incubation CQAs were identified as measurements of accelerated stability, thus allowing for the rapid identification of unstable formulations (1 week under accelerated conditions vs. 4 weeks at ambient temperature). Serum stability CQAs were identified to understand how the nanoemulsions respond to biological medium, which is critical for in vitro cell culture studies. CQAs were assessed for each run of the DoE, and values are provided in Table 2. Values that meet the CQA specification are highlighted in gray. During manufacturing, the gelation phenomenon was observed for every run, and manual stirring was necessary to continue mixing until the gelation phase ended. Despite this, each run produced a nanoemulsion with an average droplet diameter of less than 250 nm, and the majority of nanoemulsions (8 out of 11) met all CQA specifications (Table 2). Figure 5 provides a graphical summary of nanoemulsion droplet diameter (Figure 5A), PDI (Figure 5B), and diameter change after 1-week incubation at 50 °C (Figure 5C).

**Figure 6:**
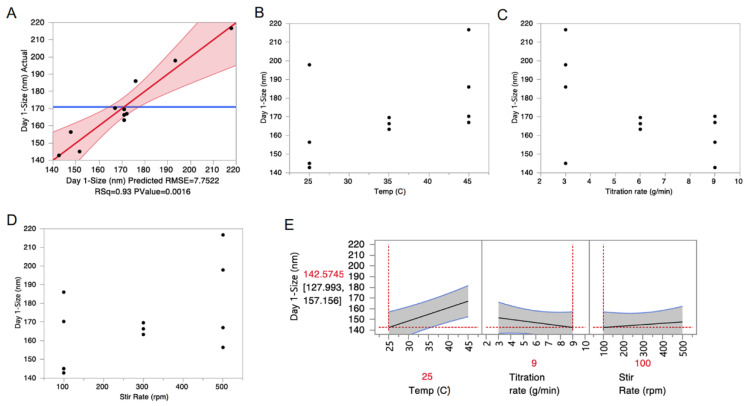
(**A**) Actual vs. predicted plot demonstrates that the multiple linear regression (MLR) model predicts the nanoemulsion diameter as a function of nanoemulsion processing parameters with an R^2^ value of 0.93. (**B**–**D**) Plots of nanoemulsion diameter vs. temperature (Panel B), water titration rate (Panel C), and stir rate (Panel D) suggest that nanoemulsion diameter decreases with increasing water titration rate, decreasing temperature, and decreasing stir rate. (**E**) The MLR prediction profiler confirms these findings.

Multiple linear regression (MLR) was used to develop a model capable of predicting nanoemulsion diameter as a function of nanoemulsion processing parameters. Such a model would be useful because, as mentioned previously, transdermal delivery is impacted by nanoemulsion droplet size. All parameters (temperature, titration rate, and stir rate) were included in the initial model, as well as all two-way interaction terms. The initial models were simplified by stepwise removal of terms with *p*-values > 0.05, and the *p*-values for each parameter included in the final model are provided in Table 3. The MLR model accurately predicts the nanoemulsion diameter (R^2^ = 0.93) as a function of titration rate, stir rate, and temperature (Figure 6A). A significant interaction was also identified between nanoemulsion stir rate and titration rate (Table 3). Parameter estimates from the MLR model indicate that increasing titration rate, decreasing stir rate, and decreasing temperature results in nanoemulsions with a smaller diameter (Table 3). Figure 6 confirms this, as average nanoemulsion diameter is smaller at 25 °C (Figure 6B), 9 g/min titration rate (Figure 6C), and 100 rpm stir rate (Figure 6D). The MLR prediction profiler (Figure 6E) shows that, at a temperature of 25 °C and a titration rate of 9 g/min, nanoemulsion diameter is not as significantly impacted by changes in stir rate. This finding is significant when considering nanoemulsion scale-up. Temperature can be well controlled at any scale, and titration rate can easily be adjusted to account for increases in scale. However, adjusting stir rate accordingly for scale-up is less straightforward, so a process that is robust to changes in stir rate would be more likely to be successfully reproduced on a different scale. For these reasons, a temperature of 25 °C, a titration rate of 9 g/min, and a stir rate of 100 rpm were selected as optimal processing conditions (DoE Run 5). Further, DoE run 5 met all CQA specifications (Table 2). A comparison of select CQAs after accelerated stability for run 5 are provided in Supplemental Figure S2. Therefore, CUR nanoemulsions were produced under these conditions on 100 g and 500 g scales.

### 3.4. Manufacture and Scale-up of CUR Nanoemulsion

**Figure 7:**
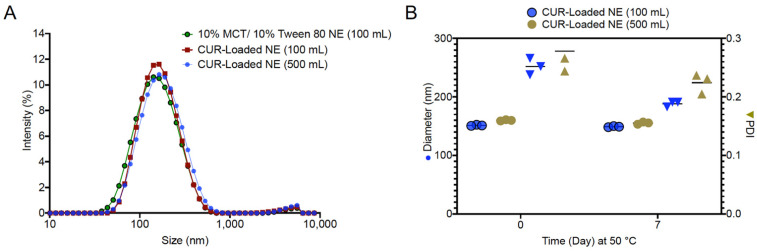
(**A**) Droplet size comparison of 10% MCT oil/10% Tween 80 nanoemulsions with and without 0.1% (*w*/*w*) CUR. CUR nanoemulsions were manufactured at 100 g and 500 g scales. (**B**) droplet diameter and PDI comparisons of CUR-loaded nanoemulsions after 1 week of storage at 50 °C.

As mentioned above, optimized processing conditions were found to be a temperature of 25 °C, 100 rpm stir rate, and 9 g/min titration rate. Therefore, CUR nanoemulsions were successfully produced at these optimized processing conditions at pilot (100 g) and scale-up (500 g) quantities. A summary of the formulation and processing parameters for these nanoemulsions is provided in Table 4, and a summary of CQA values from the two CUR-loaded emulsions is provided in Table 5. The addition of CUR to the nanoemulsion did not influence nanoemulsion formation. Droplet size of CUR nanoemulsions produced on 100 g and 500 g scales were comparable with each other, with the equivalent blank formulation produced on a 100 g scale (Figure 7A). CUR did not influence nanoemulsion colloidal stability, and a comparison of droplet diameter and PDI for the two CUR nanoemulsions after 1-week incubation at 50 °C is provided in Figure 7B. Both scales met the CQA for CUR loading, with 96.29 ± 0.76% and 95.60 ± 0.88% drug loading for the 100 g and 500 g scales, respectively. When the CUR-loaded nanoemulsions at 100 g and 500 g scales were exposed to UV light (D65 lamp), the drug-loading decreased only by 7% after 24 h exposure and only by 20% after 72 h, as measured by HPLC, supplemental information Table S2. This suggests that, upon transdermal application, the nanoemulsion retains drug loading and potential efficacy when preparation is applied every 6–8 h.

### 3.5. Cell Viability Assays

**Figure 8:**
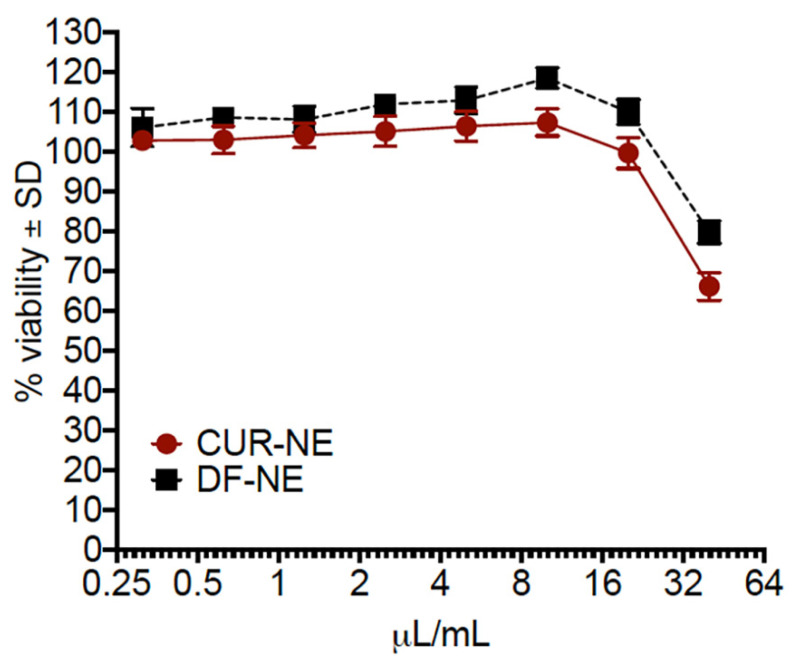
Viability of RAW 264.7 cells in the presence of CUR-loaded nanoemulsions. Nanoemulsions were diluted into the complete medium to achieve the desired nanoemulsion concentrations.

A murine macrophage cell line (RAW 264.7, American Type Culture Collection (ATCC)) was used as a model for evaluating the cellular viability of CUR nanoemulsions. These cells are not directly relevant to the product, as developed for transdermal applications, but provide the means of assessing cellular viability, since the product is easily taken up by cells exposed over the course of 6 h. In a typical experiment, the cells are exposed to CUR-loaded nanoemulsions (CUR-NE) and drug-free nanoemulsions (DF-NE) at varying concentrations (0–40 µL/mL) for 6 h, then treatment (nanoemulsion diluted in cell culture media) is removed and cell viability assayed using luminescence-based assays, as reported previously [35,36,37]. The nanoemulsions exhibited some toxicity (~20–30%) when dosed above 20 µL/mL during the 6 h period, as shown in Figure 8. Below this threshold, no toxicity was observed. There was minimal difference between the CUR-loaded and CUR-free nanoemulsions, implying that CUR itself was not the source of the increased toxicity and that Tween 80 is more likely causing toxicity at the higher dose [43]. A high surfactant concentration is appropriate for transdermal applications as it enhances drug penetration, while if applied by other routes, directly to tissues or cells (as in this assay), it can lead to saponification of the cell membrane and cell death due to membrane leakage. We also found that the highest safe dose (20 µL/mL) would be comparable to future transdermal dosing. Further studies in animals would be necessary to establish a full in vivo safety and efficacy profile in animals for transdermal applications, which are beyond the scope of this report.

### 3.6. Rheological Behavior of Curcumin Nanoemulsions

**Figure 9:**
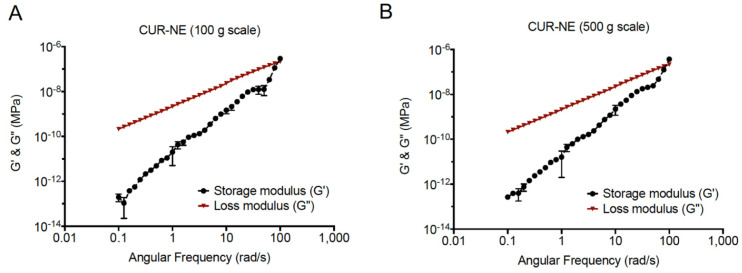
Dynamic viscoelastic curves presenting the variation of storage moduli and loss moduli with angular frequency of (**A**) 100 g CUR Nanoemulsion and (**B**) 500 g CUR Nanoemulsion at 25 °C. Data collected on DHR-20 Rheometer. Experiments ran in triplicate. Data presents average ± SD. Data was processed using the TRIOS software and plotted using Graph Pad Prism 8.

Storage moduli and loss moduli of 100 g and 500 g CUR nanoemulsions are provided in Figure 9. Storage moduli indicate the solid-like behavior of the emulsion, whereas the loss moduli is indicative of the liquid-like behavior of the emulsion. Therefore, where the loss modulus is greater than the storage modulus, liquid-like behavior dominates. For the majority of the tested angular frequencies, liquid-like behavior dominates. It is only at extremely high angular frequencies (corresponding to extremely short timescales) that solid-like behavior dominates. As a result, it is possible to say that the emulsions had good spreadability across the majority of time scales and that, at relevant timescales, the emulsion behaved as a liquid would.

## 4. Discussion

Low energy nanoemulsion formation offers significant benefits for ease of manufacturing, but efficient selection of oil and surfactant composition and SORs can prove challenging [31,32,34]. This scheme is further complicated by using appropriate surfactants to achieve the desired concentration of active pharmaceutical ingredient (API) within the nanoemulsion. In this work, we identified that CUR can be manufactured into a nanoemulsion using CPI to produce stable emulsions at the desired concentration (0.1% *w*/*w*). Although the resultant nanoemulsions exhibited acceptable CQAs, the manufacturing process was complicated by the gel intermediate observed in all evaluated formulations. The presented DoE efficiently identified that changes to the process did not mitigate the formation of the gel phase and confirmed that this phase was intrinsic to the formation of the nanoemulsion using MCT oil and Tween 80.

**Figure 10:**
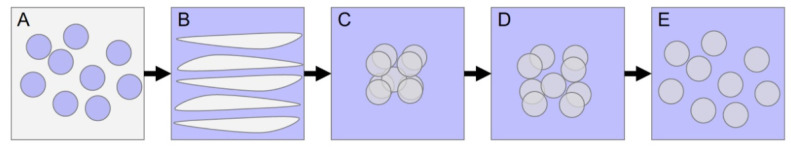
(**A**) Water is titrated into the oil/surfactant solution forming an initial w/o emulsion. (**B**) As water is continued to be added, the w/o emulsion transitions to a bicontinuous phase. (**C**) The bicontinuous phase ends as the surfactant encapsulates oil into droplets, which form a liquid crystal intermediate, resulting in gelation. (**D**,**E**) Liquid crystal breaks up as it is diluted, with additional water forming a stable o/w emulsion.

The use of the emulsion inversion point to generate nanoemulsions via CPI requires the titration of water into the oil/surfactant mixture to achieve our final formulation of 10% MCT oil/10% Tween 80/80% water. However, during the titration process, the system experiences low weight % water relative to the final nanoemulsion in the early stages due to the water addition being performed as a titration. Due to the hydrophilicity of Tween 80, the phase inversion is expected to begin prior to the oil/water ratio, reaching 1:1. The initial titration of water results in the formation of inverse micelles (IM) forming the water in oil emulsion. As the water concentration increases, the high concentration of surfactant may result in the thermodynamically favorable formation of liquid crystalline structures to minimize the interfacial surface tension. Figure 10 demonstrates the potential liquid crystal formations and mesophases that may occur as water is added to an oil/surfactant solution. Many sources claim that this transition through a liquid crystalline phase is necessary to produce stable nanoemulsions via low-energy methods [41]. These works further note that the titration rate plays a significant role in the diameter and stability of the final nanoemulsion. Maestro et al. also determined that maximum nanoemulsion stability was dependent on the amount of time spent in the cubic liquid crystal phase (oil/surfactant droplets regularly packed surrounded by water) before final dilution to nanoemulsion [42]. The DoE was designed to assess the impact of process parameters that may be adjusted at an industrial scale to mitigate the energy cost of gelation and to generate an appropriate design space to maintain the desired CQAs of the product. The results of the DoE and subsequent MLR model shows that the manufacturing scheme with the lowest temperature, the highest titration rate, and the lowest stir rate produces the highest quality nanoemulsion. Due to the impact of temperature, water titration rate and stir rate on final product CQAs are both identified as critical process parameters (CPPs).

Most low-energy emulsion studies are performed at a small scale, and there is limited information available on their scalability with respect to pharmaceuticals [32,44]. The ultimate goal of this project was to develop robust, scalable manufacturing of curcumin nanoemulsions using low-energy methods, such as CPI. The ultimate usefulness of the nanoemulsification processes is saving costs and energy while achieving a high quality final product produced on a sufficient scale. Here, we demonstrated the scalability 5-fold: from 100 to 500 g. In spite of unavoidable transition through the liquid crystalline phase and associated challenges to the current manufacturing scheme, we succeeded in producing our product on a relatively large scale. We showed that this transition step challenge can be overcome through manual intervention, while in an industrial setting, these challenges could be overcome with different impellor geometries. In earlier studies, we employed QbD strategies and MLR on microemulsions and high energy processed nanoemulsions [32,33,36]. This was the first time in which the CPI process for the production of nanoemulsification of curcumin delivery was optimized using QbD strategies. The scalability of the developed CUR-loaded nanoemulsions also highlights that the strength of presented QbD implementation is highly beneficial, leading to a relatively small number of design points needed to achieve a scalable product.

Nanoemulsions presented here are expected to increase transdermal permeability by disruption of stratum cornea lipid bilayer via surfactant penetration, enhanced intracellular delivery, enhanced penetration of intercellular pathways via droplet size reduction, and binding of positive charge nanoemulsions to negatively charged skin, increasing the likelihood of drug delivery [45]. This strategy would allow deeper penetration of CUR, leading to more effective anti-inflammatory action in the skin. Further, MCT oil and Tween 80 are commonly used pharmaceutical excipients with approved products containing higher weight content of both excipients available on the market [46]. With the cost of new pharmaceuticals continuing to fall under scrutiny, a turn to natural and generic products has the potential to fill this demand. As natural products are identified, QbD approaches, such as those presented in this work, can be utilized to transition these molecules from the lab to the market. As has been the case with CUR, although its physicochemical properties may, at first glance, severely limit its effectiveness, careful formulation development can overcome these challenges and broaden the available products for patients.

## 5. Conclusions

A CUR-loaded nanoemulsion has the potential to fill an unmet medical need for patients. Low-energy methods would allow for a more flexible manufacturing train than high pressure methods while maintaining stability and tightly controlled CQAs. A 10/10/80 (% *w*/*w*) MCT oil/Tween 80/water formulation was shown to achieve a stable nanoemulsion using the CPI method. During the development process, it was found that the Tween 80 in the formulation caused gelation during the water titration, resulting in a highly viscous cubic liquid crystal phase that could be dissolved into the aqueous phase, forming a stable nanoemulsion. This phase presents challenges to the current manufacturing scheme, which has been overcome through manual intervention to date; however, upon scale-up, the implementation of anchor blade geometry overhead mixers would allow for satisfactory control over the process. Future work will continue to study the gel intermediate phase, as well as execute process optimization studies using DoE and monitoring long-term and accelerated stability.

## Figures and Tables

**Table 1:** DoE for processing conditions of selected formulation.

DOE Runs	MCT Oil (g)	Tween 80 (g)	Water (g)	Temperature (°C)	Titration Rate (g/min)	Stir Rate (rpm)
1	10	10	80	25	3	100
2	10	10	80	45	9	500
3	10	10	80	25	9	500
4	10	10	80	35	6	300
5	10	10	80	25	9	100
6	10	10	80	45	3	100
7	10	10	80	45	9	100
8	10	10	80	25	3	500
9	10	10	80	35	6	300
10	10	10	80	45	3	500
11	10	10	80	35	6	300

**Table 2:** Summary of CQA specification testing for DoE runs.

		CQA (Specification)							
Run	Appearance	Diameter (≤250 nm)	PDI (≤0.25)	Centrifugation Diameter Change (≤10%)	Centrifugation PDI (≤0.25)	1 W 50 °C Diameter Change (≤10%)	1 W 50 °C PDI (≤0.25)	Cell Culture Diameter Change (≤10%)	Cell Culture PDI (≤0.30)
1	Homogenous	145.03	0.200	0.712	0.183	6.160	0.152	9.400	0.212
2	Homogenous	166.97	0.247	2.815	0.228	0.299	0.229	1.717	0.251
3	Homogenous	156.37	0.278	3.176	0.259	2.558	0.224	3.667	0.316
4 *	Homogenous	163.30	0.235	6.144	0.238	2.633	0.182	2.082	0.264
5	Homogenous	142.80	0.250	4.552	0.220	3.151	0.190	3.058	0.265
6	Homogenous	186.00	0.263	4.767	0.233	1.918	0.249	0.197	0.245
7	Homogenous	170.27	0.236	4.150	0.220	3.426	0.219	0.078	0.240
8	Homogenous	197.90	0.225	3.672	0.191	0.421	0.170	5.087	0.221
9 *	Homogenous	166.33	0.247	3.146	0.226	1.303	0.171	1.202	0.264
10	Homogenous	216.70	0.245	4.507	0.313	0.108	0.237	1.615	0.232
11 *	Homogenous	169.60	0.243	4.363	0.223	0.432	0.184	0.649	0.266

Values that meet the CQA specification are highlighted in grey. * Center point runs.

**Table 3:** Model parameters, estimates, standard error, and *p*-values for all terms included in the multiple linear regression model.

Source	Estimate	Standard Error	*p*-Value
Intercept	137.93	12.02	<0.0001
Titration rate (g/min)	−4.5508	0.9136	0.0025
Temp (°C)	1.2230	0.2740	0.0043
Stir Rate (rpm)	0.0587	0.0137	0.0052
Titration rate (g/min) * Stir Rate (rpm)	−0.0153	0.0046	0.0156

**Table 4:** CUR-loaded nanoemulsion and scale-up: Formulations and processing parameters.

**Formulation Composition**	**DoE Scale-100 g**	**Scale-up-500 g**
MCT Oil (g)	10.0	50.0
Tween 80 (g)	10.0	50.0
Curcumin (g)	0.1	0.5
Water (g)	79.9	399.5
**Processing Parameters**	**DoE Scale-100 g**	**Scale-up-500 g**
Temperature (°C)	25	25
Titration Rate (g/min)	9	45
Stir Rate (rpm)	100	300

**Table 5:** Summary of CQA specification testing for CUR-loaded nanoemulsion and scale-up.

	CQA (Specification)								
Scale (g)	Diameter (≤250 nm)	PDI (≤0.25)	CUR Loading (≥90%)	Centrifugation Diameter Change (≤10%)	Centrifugation PDI (≤0.25)	1 W 50 °C Diameter Change (≤10%)	1 W 50 °C PDI (≤0.25)	Cell Culture Diameter Change (≤10%)	Cell Culture PDI (≤0.30)
100	148.73	0.22	96.29 ± 0.76%	3.78	0.21	1.30	0.25	3.91	0.23
500	155.10	0.25	95.60 ± 0.88%	4.89	0.23	0.33	0.27	4.85	0.26

## Data Availability

All relevant data is included in the manuscript and will not be otherwise publicly available.

## Supplementary Materials

The following are available online at https://www.mdpi.com/article/10.3390/pharmaceutics13060880/s1, Figure S1: Solubility evaluations of curcumin in oils and surfactants selected for preformulation studies. Figure S2: (A,B) Droplet size comparison of 10% MCT/ 10% Tween 80 nanoemulsions after centrifugation or storage at 50 °C, respectively. (C,D) Droplet diameter and PDI comparisons of CUR loaded nanoemulsions after centrifugation or storage 1 week storage at 50 °C, respectively. Table S1: Initial Formulations and Processing Conditions. Table S2: Photostability of CUR nanoemulsions produced on 100 g and 500 g scales. Nanoemulsions were exposed to UV light (D65 lamp) for up to 72 h. CUR loading decreases by a rate of approximately 7% every 24 h. This suggests that the product would be appropriate for transdermal application.
