# Supplementary material for: Quality by Design Methodology Applied to Process Optimization and Scale up of Curcumin Nanoemulsions Produced by Catastrophic Phase Inversion
Source: Pharmaceutics. 2021 Jun 15;13(6):880. doi: 10.3390/pharmaceutics13060880 (PMC8232217; doi:10.3390/pharmaceutics13060880)
Supplement: Supplementary file 1 [file pharmaceutics-13-00880-s001.zip › pharmaceutics-1241774-SI.pdf]

pharmaceutics

MDPI

Supplementary Materials: Quality by Design Methodology Applied to Process Optimization and Scale up of Curcumin Nanoemulsions Produced by Catastrophic Phase Inversion

Sandeep Kumar Reddy Adena, Michele Herneisey, Eric Pierce, Paul R. Hartmeier, Suneera Adlakha, Marco A. I. Hosfeld, James K. Drennen, and Jelena M. Janjic

Table S1. Initial Formulations and Processing Conditions.

Lot	Formulation Composition						Process Conditions			
(EPPH-001)	MCT oil (g)		Tween 80 (g)		H_2O (g)		Water Titration Rate (g/min)	Vessel Geometry	Stir Rate (rpm)	Temp.
	%w/w	g	%w/w	g	%w/w	g				
-005-3	10.0	10.0	8.0	8.0	78.0	78.0	10.0	Wide 250 mL glass beaker	250	RT
-005-4	10.0	10.0	10.0	10.0	80.0	80.0				
-002-1	20.0	20.0	15.0	15.0	65.0	65.0				
-005-1	20.0	20.0	16.0	16.0	64.0	64.0				
-005-2	20.0	20.0	18.0	18.0	62.0	62.0				
-002-2	20.0	20.0	20.0	20.0	60.0	60.0				

.

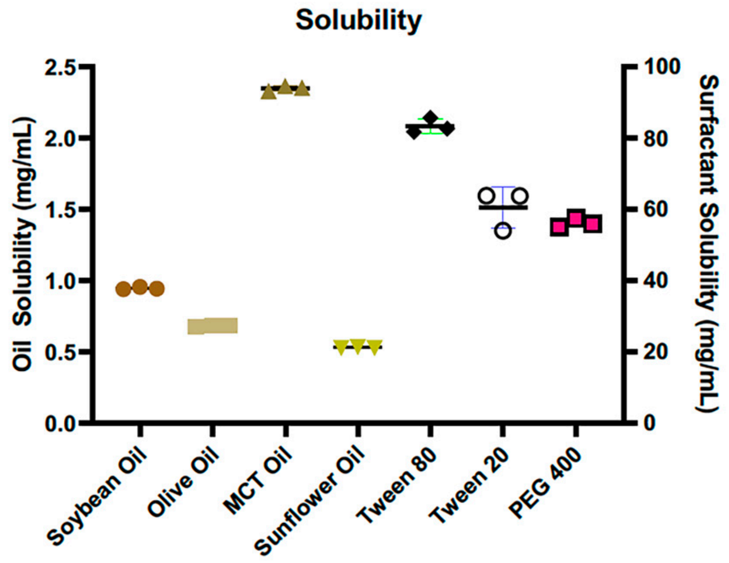

Oil/Surfactant	Soybean Oil	Olive Oil	MCT Oil	Sunflower Oil	Tween 80	Tween 20	PEG 400
Avg Solubility ± Std Dev (mg/mL)	0.95 ± 0.01	0.68 ± 0.00	2.35 ± 0.01	0.53 ± 0.00	83.43 ± 1.69	60.51 ± 4.61	56.14 ± 1.02

.

Figure S1. Solubility evaluations of curcumin in oils and surfactants selected for preformulation studies.

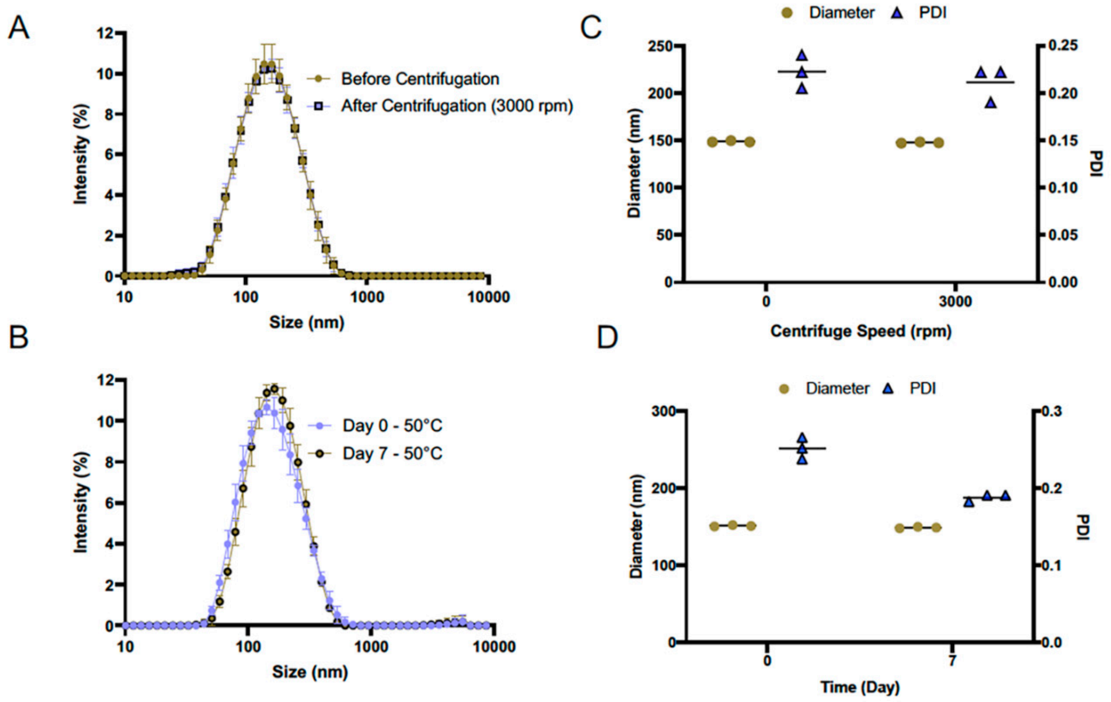

Figure S2. A-B) Droplet size comparison of 10% MCT/ 10% Tween 80 nanoemulsions after centrifugation or storage at 50 °C, respectively. **C-D)** Droplet diameter and PDI comparisons of CUR loaded nanoemulsions after centrifugation or storage 1 week storage at 50 °C, respectively.

Table S2. Photostability of CUR nanoemulsions produced on 100g and 500g scales. Nanoemulsions were exposed to UV light (D65 lamp) for up to 72h. CUR loading decreases by a rate of approximately 7% every 24h. This suggests that the product would be appropriate for transdermal application.

	**Manufacturing Scale**	**Concentration (μg/mL)**	**% Loading**
Baseline	100g	967.95±7.64	96.29±0.76
	500g	956.53±8.85	95.60±0.88
24h, D65 Exposure	100g	893.61±2.59	88.90±0.26
	500g	889.88±7.30	88.93±0.73
72h, D65 Exposure	100g	756.03±2.08	75.21±0.21
	500g	762.77±16.73	76.23±1.67
